# Expression of Intratumoral IGF-II Is Regulated by the Gene Imprinting Status in Triple Negative Breast Cancer from Vietnamese Patients

**DOI:** 10.1155/2015/401851

**Published:** 2015-09-10

**Authors:** Vinodh Kumar Radhakrishnan, Lorraine Christine Hernandez, Kendra Anderson, Qianwei Tan, Marino De León, Daisy D. De León

**Affiliations:** Center for Health Disparities and Molecular Medicine, School of Medicine, Loma Linda University, Loma Linda, CA 92350, USA

## Abstract

African American women suffer higher incidence and mortality of triple negative breast cancer (TNBC) than Caucasian women. TNBC is very aggressive, causing the worst clinical outcome. We previously demonstrated that tumors from these patients express high IGF-II and exhibit high activation of the IGF signaling pathways. IGF-II gene expression is imprinted (monoallelic), promotes tumor progression, and metastasis and regulates Survivin, a TNBC prognostic marker. Since BC mortality has increased among young Vietnamese women, we analyzed 48 (paired) TNBC samples from Vietnamese patients to assess IGF-II expression. We analyzed all samples by qrtPCR for identification of IGF-II heterozygosity and to determine allelic expression of the IGF-II gene. We also analyzed the tissues for proIGF-II and Survivin by RT-PCR and Western blotting. A total of 28 samples displayed IGF-II heterozygosity of which 78% were biallelic. Tumors with biallelic IGF-II gene expression exhibited the highest levels of proIGF-II and Survivin. Although 100% of these tissues corresponding normal samples were biallelic, they expressed significantly lower levels of or no proIGF-II and Survivin. Thus, IGF-II biallelic gene expression is differentially regulated in normal versus tumor tissues. We propose that intratumoral proIGF-II is dependent on the IGF-II gene imprinting status and it will promote a more aggressive TNBC.

## 1. Introduction

IGF-II is expressed in breast cancer and “free” circulating IGF-II levels in humans are correlated to breast tumor size [1]. The expression of the IGF-II gene is tightly regulated and inhibited by tumor suppressor genes such as PTEN and p53 [2, 3]. IGF-II is positively regulated by estrogen, progesterone, prolactin, and growth hormones in normal breast development and in breast cancer progression [4–6]. Thus, IGF-II expression is important in normal breast development and increased IGF-II levels in the mammary gland contribute to tumor progression.

IGF-II was the first gene discovered to be imprinted and it is expressed exclusively from the paternal allele in humans [7]. Genomic imprinting is an important biological phenomenon defined by allelic-specific epigenetic modifications unique to the parent-of-origin specific gene expression [8]. IGF-II is also the first imprinted gene found to display loss of imprinting (LOI) or aberrant imprinting in human diseases [9]. In addition, LOI of the IGF-II gene is a frequent alteration in breast cancer [10]. The imprinting of the IGF-II gene is tissue specific [11] and it is predominantly monoallelic, except for certain regions in the brain where biallelic expression is normal [12].

Activation of the normally silent maternal allele of IGF-II is considered an early event in tumor development. Other genetic changes in the IGF-II gene such as Single Nucleotide Polymorphisms (SNPs) can influence the risk and/or severity of many diseases and may also be associated with increased risk of cancer. IGF-II ApaI (rs680) has been widely used to assess IGF-II loss of imprinting [13]. In our present study we use IGF-II rs680 SNP to determine loss of imprinting and, in addition, we will assess the effect of this SNP on proIGF-II protein expression.

TNBC patients are usually younger than 40 years old, have lower survival rates, and face worse clinical outcomes when treated with chemotherapy [14, 15]. This aggressive form of BC represents 15% of all invasive BC cases, yet it disproportionally affects AA women, who represent over one-third of this BC group. We have demonstrated that breast cancer tumors from AA patients express significantly higher levels of IGF-II when compared to tumors from Caucasian American women (CA) [16]. Our studies also showed that these tumors display high levels of activation of the IGF signaling pathways and antiapoptotic proteins such as Survivin which are associated with a more aggressive and chemoresistant form of the disease.

TNBC is increasing among younger Vietnamese women; however, it is not known which factors contribute to this condition. Understanding the growing evidence of the critical role of IGF-II in cancer, we thought to determine whether IGF-II gene regulation is present in TNBC among Vietnamese women. We used the IGF-II rs680 SNP to determine the IGF-II imprinting status in these tissues [17] and to determine if this genetic alteration is associated with TNBC in this ethnic group.

With hormone-based therapies, treatment pivots on whether a given hormone is recognized by its receptor [18]. Nevertheless, TNBC tumors do not express the traditional hormone receptors targeted for treatment: estrogen, progesterone, and Her2 receptors. The identification of alternate prognostic markers that can be targeted for therapy may result in new treatment of TNBC [19]. Thus, the present study also assessed how IGF-II imprinting regulates the expression of Survivin and related proteins associated with IGF-II signaling in the development and progression of breast cancer.

## 2. Materials and Methods

### 2.1. Patient Samples and Information

Frozen paired TNBC tissue samples (normal/malignant) were commercially obtained from ILSbio (Integrated Laboratory Services-Biotech, 100 Radcliffe Drive, Chestertown, MD 21620 USA) and stored at −80°C. ILSbio randomly obtained the samples from Vietnamese breast cancer patients residing in the USA. Each specimen comes with detailed clinical information and pathological reports. The diagnosis is confirmed by at least three pathologists. The pathology reports contain the basic information of patients and the verified characterization of normal and tumor tissues (Table 1). All ILSbio specimens are collected under Institutional Review Board (IRB) approved protocols, ensuring that strict ethical guidelines are followed to protect patient confidentiality and safety. Each sample has the patients consent for use in a wide range of research including the development of commercial products or services. The samples are identifiable only by barcodes to ensure an unbiased assessment as well as patient confidentiality.

#### 2.1.1. gDNA qrtPCR to Determine IGF-II Heterozygosity

High molecular weight DNA was extracted by conventional methods using the genomic DNA extraction from Invitrogen. DNA PCR reactions were performed on 100 ng of genomic DNA essentially using forward primer 5′CTTGGACTTTGAGTCAAATTGG-3′ and reverse primer 5′-GGTCGTGCCAATTACATTTCA-3′ which flank a transcribed ApaI polymorphism in Exon 9 of the IGF-II gene to differentiate the distinct IGF-II allelic profile. The resultant 292 bp DNA products were digested with ApaI and electrophoresed in a 3% agarose gel and stained with ethidium bromide. Digested samples yielding bands of 292, 227, and 65 bp were designated Heterozygous (Het), and samples yielding only the 292 bp size band are Homozygous (Hom) while samples yielding 227 and 65 bp are Homozygous with SNP (Hom SNP). IGF-II allelic sequences are checked for and confirmed by applied biosystems sequencing and analyzed by sequence scanners for the IGF-II Exon 9 region. The sequencing is performed by Eton Biosciences for identification of the IGF-II Exon 9.

#### 2.1.2. nPCR to Determine IGF-II Allelic Expression

Total RNA was extracted using TRI Reagent (Molecular Research Center, Cincinnati, OH) in combination with Zymo RNA extraction and purification kit. RNA was purified according to the manufacturer's Direct-zol RNA MiniPrep protocol. To eliminate contaminating DNA, the samples were treated with DNAse prior to reverse transcription and subsequent RT-PCR amplification. One *μ*g of RNA was amplified with high efficiency utilizing random primer to cDNA (Reverse Transcription System, Bio-RAD). A reverse transcription PCR (RT–PCR) was performed in 10 *μ*L of first-strand cDNA with forward primer 5′-TCCTGGAGACGTACTGTGCTA-3′ and reverse primer 5′-GGTCGTGCCAATTACATTTCA-3′. The PCR cycling conditions were the same as described above for gDNA PCR and it yields a 1.12 kb IGF-II product confirmed by agarose gels. DNA-specific PCR product (1.4 kb) could be distinguished from the cDNA-specific product (1.12 kb). None of the RT-PCR preparations yielded a 1.4 kb genomic DNA band, indicating that there was no DNA contamination and that DNAse treatment was successful. To increase the 1.12 kb band we used nested PCR (nPCR) on the 1.12 kb PCR products using forward primer 5′CTTGGACTTTGAGTCAAATTGG-3′ and reverse primer 5′-GGTCGTGCCAATTACATTTCA-3′ to generate a shorter RT-PCR fragment of 292 bp harboring the ApaI polymorphic site as previously determined in the gDNA. The gDNA PCR product for all 48 samples was loaded onto 3% agarose gels and stained with ethidium bromide. The agarose gel bands were visualized under UV light to determine the IGF-II allelic pattern. ApaI restriction digestion enzyme analysis yielding bands of 292, 227, and 65 bp were designated biallelic samples (BA), those that yielded only the 292 bp were designated as monoallelic (MA), and samples yielding 227 and 65 bp are monoallelic with SNP (MAS).

#### 2.1.3. RNA, cDNA, QPCR, and Conventional Gels

Total RNA was extracted using TRI Reagent (Molecular Research Center, Cincinnati, OH) according to the manufacturer's protocol. 50 mg of frozen tissue was cut and homogenized with the next advance motor drive using cool air circulating system. Samples were treated with Ambion DNAse to get rid of genomic DNA contamination. The total RNA stock was kept at −80°C until assayed. The cDNA was synthesized using iScript cDNA Synthesis Kit from Bio-Rad. The genes of interest (GOI) analyzed were IGF-I, IGF-II, Survivin, IGF-IR, IGF-IIR, IRA, IRB, ESR-*α*, ERBB2/Her2, PRLR, and VEGFR. The primers, forward (F) and reverse (R), for the above GOI were designed using intron spanning assay. 18s rRNA was used as housekeeping reference gene to calculate the fold mRNA change. The analysis was performed using the CFX 1000 QPCR machine. The results were analyzed using the CFX Manager version 1.0. Qbase and GraphPad Prism 5 software were used to quantitate the relative mRNA fold expression change for the above GOI.

### 2.2. Tissue Protein Extraction and Western Blot

Tissue cell lysates (50–75 mg) were prepared in RIPA buffer (1X TBS, 1% Nonidet P-40, 0.5% sodium deoxycholate, 0.1% SDS, 0.004% sodium azide, 10 *μ*L/mL protease inhibitor cocktail, and 10 *μ*L/mL sodium orthovanadate). A total of 90 *μ*g was loaded into polyacrylamide-SDS gradient gels (4%–12%), transferred to a PVDF membrane (Invitrogen, Carlsbad, CA) using X-cell Sure-Lock electrophoretic transfer module (Invitrogen, Carlsbad, CA). Protein concentration was measured using the BCA Protein Assay Kit from Thermo Scientific (Rockford, IL). PVDF membranes were blocked in PBS/0.05% Tween and 3% milk for 1.5 hr at 4°C followed by 3x washes in PBS during 10 minutes each. Membranes were then incubated with IGF-II monoclonal antibody (Amano, 1 : 2500 dilution) in 3% BSA overnight. Membranes were washed three times for 10 min each in PBS 0.05% Tween. Following the washes, the membranes were incubated with the corresponding biotinylated anti-mouse antibody (IgG (H + L) vector BA-9200). Protein visualization was achieved by using enhanced chemiluminescence (ECL) and autoradiography with Hyperfilm ECL film (Amersham, Arlington Heights, IL). The signals on the X-ray films were quantified using ChemiImager 4000 (Alpha Innotech Corporation). Ponceau red was used to stain the Western blots and to normalize for loading control. Ponceau red stain was chosen among a limited number of alternatives recommended to normalize sample loading [20, 21].

### 2.3. Statistical Analysis

Statistical differences between mean values were determined by using one-way ANOVA and Wilcoxon Signed Rank Test comparing the paired normal/malignant samples in GraphPad Prism 5. Values are expressed as the mean ± SEM of two or more replicate experiments. A level of ^*∗*^ *P* < 0.05 was considered significant.

## 3. Results

### 3.1. IGF-II Allelic Status in TNBC Samples from Vietnamese Patients

A total of 48 informative random samples (24 paired normal/malignant breast tissue samples) of Vietnamese (VIET) TNBC patients are described in Table 1. These samples were analyzed to identify heterozygosity and to select the Heterozygous (Het) samples for further testing to identify the imprinting status of the IGF-II gene.

**Figure 1:**
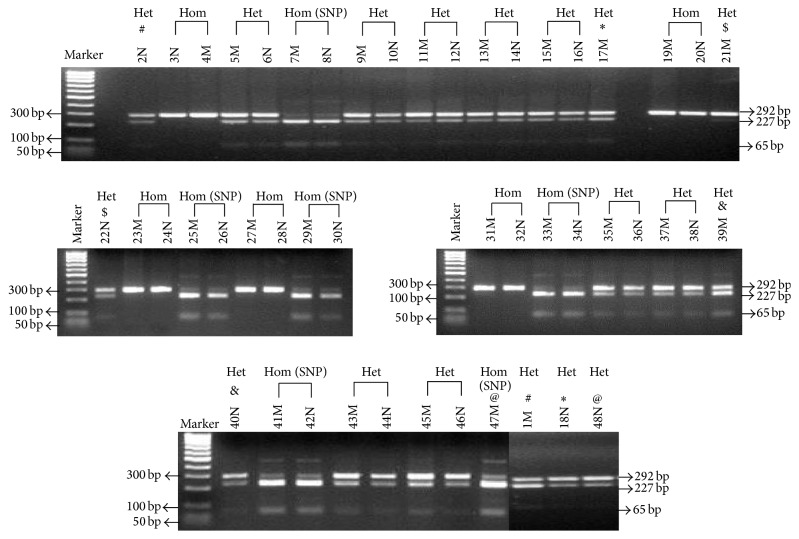
3% agarose gel showing ApaI restriction digestion of the 292 gDNA PCR products for the 48 breast normal (N) and malignant (M) samples. The IGF-II allelic pattern determined to be Heterozygous (Het; *n* = 27) displayed three bands of 292, 227, and 65 bp. The Homozygous with SNP (Hom SNP; *n* = 11) allelic pattern displayed two bands of 227 and 65 bp. Only one band (292 bp) is seen in the Homozygous without a SNP (Hom; *n* = 10) IGF-II allelic pattern. Symbols #, *∗*, $, &, and @ represent paired samples from normal and malignant that were not next to each other's lane.

Figure 1 shows a composite of 5 representative 3% agarose gels of all 48 TNBC samples. Symbols #, *∗*, $, &, and @ were used to identify paired samples that were not loaded next to each other. The Heterozygous profile for IGF-II was identified by the presence of two polymorphic bands of 292 bp (undigested) and 227 bp + 65 bp if the IGF-II rs680 SNP is present (digested). The Homozygous profile without SNP was identified by the 292 bp single band and Homozygous with SNP yielded two fragments 227 bp + 65 bp, respectively. Samples were further identified by N (normal tissue) and M (malignant tissue). Thus, the gDNA analysis of all 48 TNBC samples resulted in samples characterized as Heterozygous (*n* = 27), Homozygous (*n* = 10), and Homozygous with a SNP (*n* = 11). Thus, approximately 58% of the samples were Het, 21% were Hom, and another 21% were Hom SNP. Therefore, approximately 80% of all samples express the IGF-II rs680 SNP. The parental origin of the SNP was not considered in these experiments since the parental blood samples were not available for analysis.

### 3.2. Biallelic Expression of IGF-II in VIET TNBC Patients

**Figure 2:**
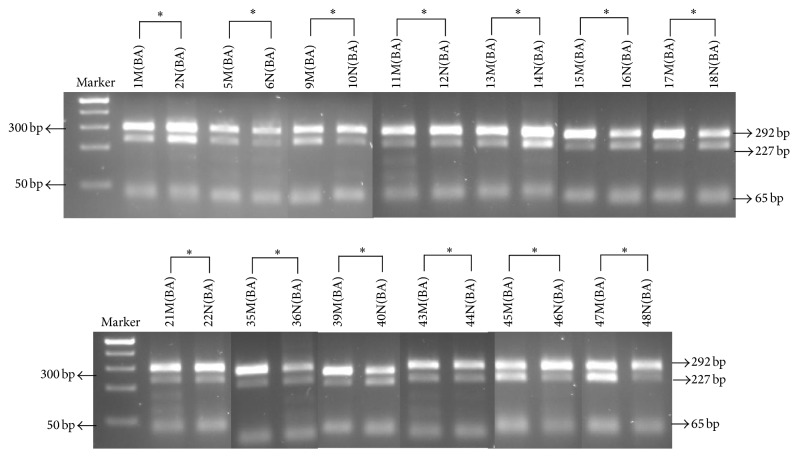
ApaI digestion of the 292 bp nested PCR (QPCR) fragment generated from the fragment of 1.12 RT-PCR reactions. Biallelic expression was identifiable by the presence of each of the 292, 227, and 63 bp restriction fragments. First lane shows Fisher's exACTGene 100 bp DNA Ladder, showing the 500 bp–25 bp respective molecular weight size markers.

To analyze imprinting of the IGF-II gene, we selected the samples that were Heterozygous (Het) for the transcribed ApaI polymorphism. A total of 26 Het samples were analyzed to identify allelic expression of the IGF-II gene (Figure 2). Surprisingly, all 26 Het samples (13N + 13M) analyzed were biallelic (BA). Thus, 100% of the Het normal paired samples were BA.

### 3.3. IGF-II rs680 Single Nucleotide Polymorphism (SNP) Sequencing

**Figure 3:**
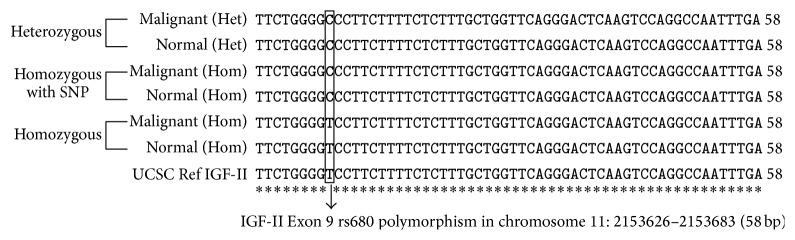
Representative standard sequencing alignment for IGF-II Exon 9 region chromosome 11: 2153626–2153683 (58 bp). The sequenced samples show that the Heterozygous (Het) and the Homozygous with SNP tissues have the (C) cytosine and the Homozygous samples have the (T) Thymidine in reference to the USCS genome Feb. 2009 (GRCh37/hg19) released version as reference gene.

The gDNA PCR products of the TNBC samples were sequenced to show the rs680 SNP with reference USCS genome browser Feb. 2009 (GRCh37/hg19) released version for IGF-II Exon 9. The sequenced samples show that the BA and the MAS tissues have the (C) cytosine and the MA samples have the (T) Thymidine in reference to the human genome web browser (http://genome.ucsc.edu/). This sequence verified the IGF-II allelic pattern and confirmed the gDNA ApaI restriction enzyme digestion pattern as shown in Figures 1 and 3. The samples were sequenced using the reverse primer 5′-GGTCGTGCCAATTACATTTCA-3′ to identify the allelic pattern for IGF-II at Exon 9 and aligned by EMBI Clustal W software.

### 3.4. Expression of proIGF-II (17.5 kDa) and Survivin (16.5 kDa) Proteins

**Figure 4:**
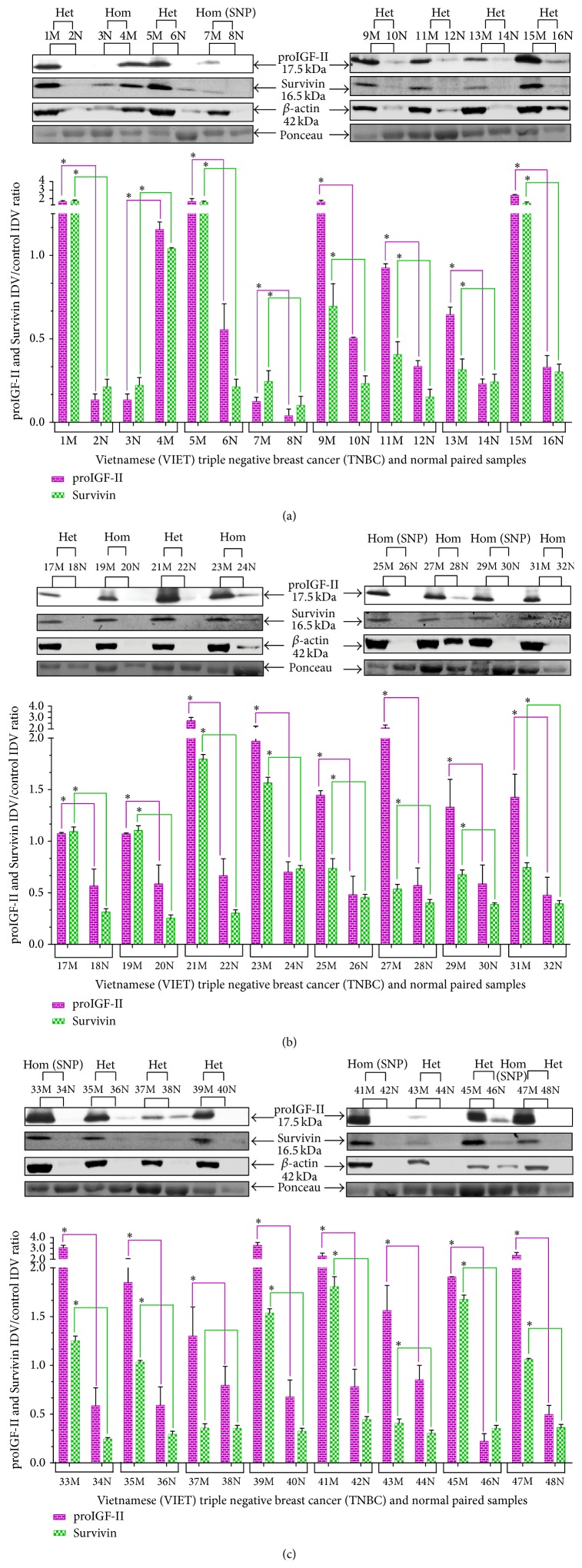
((a), (b), and (c)) Western blot of paired normal/malignant TNBC samples (1–48). Total number of samples (*n*) analyzed per group was as follows: Heterozygous (Het; *n* = 27), Homozygous with SNP (Hom SNP; *n* = 11), and Homozygous (Hom; *n* = 10). Bar graphs (a–c) of free proIGF-II (17.5 kDa) and Survivin (16.5 kDa). Ponceau red staining was used to normalize for sample loading. Bars represent the mean ± SE of all normalized samples per group. Asterisks indicate values statistically significant ^*∗*^(*p* < 0.05) using the Wilcoxon paired *t*-test.

**Figure 5:**
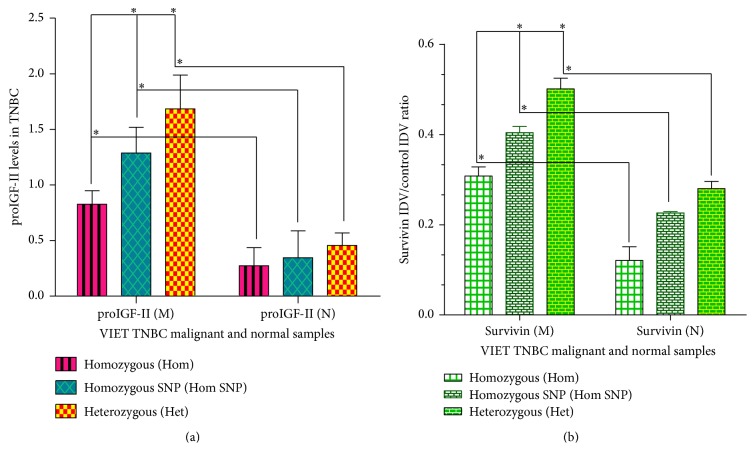
(a) Representative bar graph of proIGF-II (17.5 kDa) in paired malignant and normal TNBC samples in Hom, Hom SNP, and Het. Figure depicts the averages of three separate experiments and each was done in triplicate. Heterozygous (N = 14, M = 13), Homozygous with SNP (N = 5, M = 6), and Homozygous (N = 5, M = 5). Asterisks indicate values statistically significant ^*∗*^(*p* < 0.05) using the Wilcoxon paired *t*-test. (b) Representative bar graph of Survivin (16.5 kDa) in paired malignant and normal TNBC samples in Hom, Hom SNP, and Het. Figure depicts the averages of three separate experiments and each was done in triplicate. Heterozygous (N = 14, M = 13), Homozygous with SNP (N = 5, M = 6), and Homozygous (N = 5, M = 5). Asterisks indicate values statistically significant ^*∗*^(*p* < 0.05) using the Wilcoxon paired *t*-test.

We analyzed the levels of proIGF-II and Survivin protein in all 24 paired informative cases of TNBC by Western blot. Bands were revealed using chemiluminescence followed by exposure to radiography film. Relative protein quantification was determined by using QuantityOne 1-D Analysis Software and data is expressed as IDV proIGF-II and Survivin units/control IDV protein units from Ponceau red stain as seen in the *y*-axis in Figures 4(a), 4(b), 4(c), 5(a), and 5(b).

The use of *β*-actin and many other standard control proteins used to normalize loading was attempted unsuccessfully. The housekeeping gene *β*-Actin (42 kDa) was initially probed to be used as a loading control but, as seen, normal tissues expressed very low *β*-Actin levels when compared to their respective malignant samples (Figures 4(a) and 5(b)). The Western blot figures show *β*-actin bands and demonstrate the limitation of its use as a loading control when analyzing normal and tumor tissues. Note that *β*-actin is almost undetectable in normal (N) breast tissue samples; in contrast, a strong 60 kDa band signal is seen in the same lanes stained with Ponceau red demonstrating the presence of protein. *β*-actin is essential in cell proliferation and it is a critical component of the cancer cell cytoskeleton [22]; thus it is an unreliable loading control to use in our study. Ponceau red stain was chosen among a limited number of alternatives recommended to normalize sample loading [20, 21].

Overall, as seen in Figures 5(a) and 5(b), the malignant tissue samples (M) expressed higher levels of proIGF-II and Survivin when compared to the normal breast (N) samples that express little or no proIGF-II and Survivin (*P* ^*∗*^ < 0.05). The levels of proIGF-II and Survivin were highest in the Heterozygous (BA) samples (Het; *n* = 14) ≫ compared to Homozygous with SNP samples (Hom SNP; *n* = 5) > which were higher than Homozygous tissue samples that have no SNP (Hom; *n* = 5). Tumors with BA IGF-II gene expression exhibited the highest levels of proIGF-II and Survivin. Although 100% of these tissues corresponding normal samples were BA, they expressed significantly lower levels or no proIGF-II and Survivin. Thus, IGF-II protein expression is differentially regulated in normal versus tumor tissues.

### 3.5. Quantitative RT-PCR Analysis of Selected Gene Expression in TNBC

**Figure 6:**
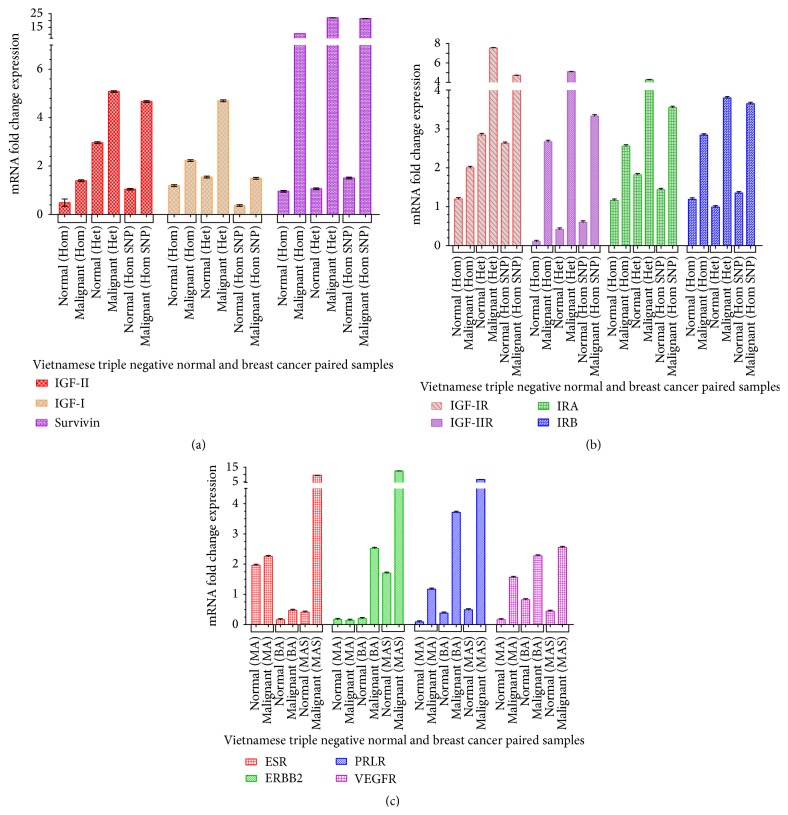
(a) Representative bar graph of IGF-II, IGF-I, and Survivin mRNA fold levels from 3 different patient samples ran in three separate qrtPCR assays done in triplicate. (b) Representative bar graph of Insulin-Like Growth Factor-I Receptor (IGF-1R), Insulin-Like Growth Factor-II Receptor (IGF-IIR), Insulin Receptor A (IRA) and Insulin Receptor B (IRB) mRNA fold levels from 3 different patient samples ran in three separate qrtPCR assays done in triplicate. (c) Representative bar graph of Estrogen Receptor-*α* (ESR-*α*), Human Epidermal Growth factor Receptor 2 (ERBB2/Her2), Prolactin Receptor (PRLR), and Vascular Endothelial Growth Factor Receptor (VEGFR) mRNA fold levels from 3 different patient samples ran in three separate qrtPCR assays done in triplicate.

Representative 9 paired samples were selected based on their mRNA quality and were analyzed by quantitative RT-PCR to assess the gene expression of IGF-II, IGF-I, Survivin (Figure 6(a)), IGF-IR, IGF-IIR, Insulin Receptor A (IRA), Insulin Receptor B (IRB) (Figure 6(b)), Estrogen Receptor-*α* (ESR-*α*), Human Epidermal Growth Factor Receptor 2 (ERBB2/Her2), Prolactin Receptor (PRLR), and the Vascular Endothelial Growth Factor Receptor (VEGFR) (Figure 6(c)). The 18S rRNA was used to normalize mRNA fold change expression (CFX Bio-Rad Manager, qbase software and GraphPad Prism 5). The data show that normal breast tissues (N) have lower levels of IGF-II, IGF-I, Survivin, and related receptors IGF-IIR, IRA, IRB, ESR-*α*, ERBB2, PRLR, and VEGFR when compared to the malignant breast tissues. The IGF-IIR, IRA, IRB, and VEGFR are higher in Het when compared to the Hom and Hom SNP.

## 4. Discussion

African American (AA) women have a lower incidence of BC but they have a higher occurrence of TNBC and increased mortality [23]. We demonstrated that BC tumors from AA patients have high levels of IGF-II and higher activation of the IGF signaling pathways [24]. Since young Vietnamese women are affected by TNBC [25], we designed the present study to determine the allelic regulation and expression of IGF-II in TNBC tumors from Vietnamese women. We examined the genomic imprinting status of IGF-II for specific allele expression to determine if disrupted imprinting, as evidenced by biallelic (BA) expression, is a mechanism of altered gene expression in breast cancer in this population [17, 26].

To our knowledge, this study is the first to identify free intratumoral IGF-II in TNBC paired samples and to demonstrate that BA expression of this growth factor gene results in significantly higher expression of IGF-II mRNA and free proIGF-II protein. The free proIGF-II protein levels were highest in the BA tumors followed by intermediate IGF-II levels in Homozygous (Hom) with SNP tumors and the lowest IGF-II levels were detected in the Hom without SNP tumors. Interestingly, IGF-II level in the BA normal tissues was undetectable or significantly lower than that in the BA paired tumor tissues, indicating that IGF-II expression is differentially regulated in the nontransformed tissues. Thus, loss of IGF-II imprinting precedes the malignant transformation and breast cancer development in the samples analyzed of young Vietnamese TNBC patients.

IGF-II bioavailability is a critical issue because most of the systemic IGF-II is associated with IGFBPs and those circulating levels cannot distinguish between secretion from liver, benign breast lesions, and breast cancer tumors. Therefore, rather than acting in an endocrine fashion, IGF-II produced in tumors is more likely working in a paracrine/autocrine mode. Thus, intratumoral IGF-II levels should be considered a better reflection of its potential actions in cancer growth and a more accurate estimate of IGF-II allelic dosage. Therefore, blood test for IGF-II may be inaccurate since it does not reflect true bioavailable IGF-II for tumor growth. IGF-II imprinting status will not only impact carcinogenesis but also impact critical functions regulated by this growth factor such as fetal development, cell cycle, apoptosis, and diabetes [27–29]. Interestingly, a recent review presents evidence of how loss of IGF-II imprinting can promote “cancer initiating cells” supporting the hypothesis of the epigenetic progenitor model of cancer [30].

Our previous studies of AA paired breast tumor/normal tissues demonstrated that higher levels of intratumoral IGF-II were correlated to Survivin, a critical antiapoptotic protein considered a marker of poor prognosis in TNBC [31–33]. Furthermore, in a breast cancer cell model, we showed that IGF-II stimulated Survivin and that IGF-II siRNA treatment completely abrogated the expression of this TNBC survival prognostic marker. In addition, those studies also showed that decreased IGF-II caused a significant reduction of Survivin resulting in increased sensitivity to resveratrol. Exogenous treatment with IGF-II restored the levels of Survivin in this cell model system and prevented cell apoptosis by inhibiting mitochondrial membrane depolarization [33]. The interaction between IGF-II and Survivin resulted in the protection of the mitochondria and the inhibition of apoptosis, key factors in promoting chemotherapy resistance. Resistance to currently available chemotherapy treatments is a major factor in the increased breast cancer mortality observed in TNBC patients [14, 15]. Thus, LOI of IGF-II will cause higher levels of free IGF-II promoting increased Survivin, among other proteins, and stimulating a more aggressive breast cancer progression and a breast tumor that is less responsive to chemotherapy.

It is well known that the expression of IGF-II is imprinted and regulated by methylation and, normally, only the paternal allele is transcribed [34–36]. Nevertheless, our results demonstrated that biallelic expression of IGF-II was present in 100% of the normal paired samples, thus demonstrating that “loss of imprinting” (LOI) is not the result of carcinogenesis in these samples. Since LOI resulted in higher intratumoral free IGF-II, we can expect, then, that LOI will contribute to an enhanced proliferative cell signaling milieu promoting a fast growing tumor, chemoresistance, and increased breast cancer death [17]. Other studies have also shown that IGF-II allelic dosage matters and that it is not an inconsequential genetic occurrence. Szabó and Mann showed that embryos were less likely to survive when both the paternal and the maternal IGF-II alleles were not expressed [37]. Likewise, if the silent maternal IGF-II allele is activated while the normally expressed paternal allele remains silent, abortion occurs as a result of the trophoblast cells failure to grow. Differentiation of trophoblastic cells is specifically regulated by IGF-II, not IGF-I [38]. LOI of IGF-II also occurs in 25% of Beckwith Wiedemann syndrome (BWS) as well as other tumors [9]. Since our study showed that higher levels of IGF-II are expressed in tumors carrying the rs680 SNP, it will be reasonable to assume that if carriers of this genetic variance develop cancer, they will be at a higher risk of developing a very aggressive disease. The IGF-II rs680 SNP have been associated with exercise induced muscle damage [39]. A higher consumption of dairy products is also associated with increased height depending on the rs680 IGF2 genotype [40]. No associations of the IGF-II SNP rs680 have been previously described with TNBC; thus, our study is the first one to identify this SNP which may impact the aggressiveness of this disease.

Our study also shows that increased level of IGF-II in BA and Hom SNP breast tissues correlates with higher expression of receptors important in breast cancer development and progression. Specifically, the mRNA for the receptors IRA, IRB, VEGFR, ER-*α*, PRLR, and ERBB2 expression correlate with the allelic dosage of IGF-II. This suggests the genomic imprinting of IGF-II; that is, IGF-II allelic dosage may play a critical role in tumorigenesis by triggering the progression of cells already harboring an oncogenic insult. IGF-II binds the IGF-IR and the Insulin Receptor A (IRA) to activate a series of mitogenic and antiapoptotic signaling pathways that includes AKT, MAPK, and NFKB [24, 41–43]. In addition, IGF-II binds the IGF-II receptor resulting in activation of the g protein signaling cascade and modulation of the lysosomal enzyme trafficking to regulate apoptosis, metastasis, proliferation, and motility [44–46]. Furthermore, IGF-II engages in cross talk signaling with other receptors such as the estrogen receptor [47, 48]. Indeed, our laboratory previously demonstrated that IGF-II binding to the IGF-IR and IRA promoted the phosphorylation of ER-*α* and ER-*β* independent of estrogen [49]. Of great significance, IGF-II is regulated by estrogen and progesterone and, in turn, IGF-II stimulates these steroid receptor's expression and activation [50]. These reciprocal interactions result in a positive stimulatory feedback that also amplifies the respective receptors signaling cascade [51].

High IGF-II levels are also an important factor in other endocrine conditions associated with a higher risk of breast cancer such as diabetes, metabolic syndrome, and obesity. Epidemiological studies looking at the development of type 2 diabetes and the metabolic syndrome, which are diseases highly prevalent among African American (AA) women, have shown that both diseases are strongly associated with increased breast cancer risk [52, 53]. Interestingly, we showed that significantly higher IGF-II levels and Insulin Receptor A were present in normal breast tissues of AA women leading us to propose that IGF-II was a common important link between breast cancer and diabetes in this ethnic group [41]. Overexpression of IGF-II leads to type 2 diabetes in a transgenic mouse model through a direct effect of IGF-II on *β*-cell proliferation [54]. These transgenic mice overexpressing IGF-II in *β*-cells were hyperinsulinemic early in life and showed altered glucose and insulin tolerance tests, as well as insulin resistance. Of great significance, the IGF-II E-peptide derived from the cleavage of proIGF-II regulates insulin secretion in the pancreas [55]. IGF-II is also an important factor in obesity [56]. In contrast to our observations among AA, the IGF-II levels in the normal breast tissues of Vietnamese women in the present study were low or undetectable. Likewise, there is no IGF-II relationship with diabetes and breast cancer among Vietnamese women.

LOI of IGF-II in 100% of the normal breast tissues paired to BA tumors in this study was unexpected as well as surprising. In addition to the effect of IGF-II allelic dosage, our study also showed that the presence of the rs680 IGF-II SNP occurred in approximately 80% of the Vietnamese breast tissue samples. We acknowledge that our Vietnamese paired breast sample number is small (*n* = 48); however, we have compared the allele count for the rs680 SNP in our paired breast samples from other ethnic groups (African American *n* = 36, Caucasian *n* = 32, and South Korean *n* = 42) to the 1000-Human Genome Ensembl for the same populations (http://uswest.ensembl.org) and the published percentage allele count is almost identical to ours (Supplementary Figures 1-2 in Supplementary Material available online at http://dx.doi.org/10.1155/2015/401851). Thus, the allele count of the IGF-II rs680 in our small sample size is comparable to the allele count in the reference populations where the samples range from 181 to 379 (Supplementary Figure 2).

## 5. Conclusions

Thus, we have demonstrated that LOI and the occurrence of the rs680 IGF-II SNP result in higher levels of intratumoral free IGF-II in breast cancer tumors of young Vietnamese women affected with TNBC. Given the pleiotropic effects of IGF-II and its multiple interactions with so many receptors, we propose that IGF-II be considered a potential molecular marker in the differential diagnosis and treatment of TNBC patients. Moreover, understanding how IGF-II promotes the development of a hormone-independent tumor and its progression into TNBC will be critical for designing novel and more effective targeted therapies badly needed to reduce the breast cancer survival disparity of this disease.

## Supplementary Material

Since our breast cancer samples from Vietnamese patients is limited (n=48), we provided Supplementary Figure 1 which represents the results of the IGF-II rs680 C/T allele count from the 1000 Human Genome Ensemble () from different ethnic groups.
Supplementary Figure 2 represents unpublished data from our own IGF-II rs680 C/T allele count from paired breast cancer samples from Caucasian American (n=32), South Korean (n=42) and African American (n=36).
Please note that when we compared the allele count for the rs680 SNP in our paired breast sample analysis from Caucasian American, South Korean and African American (Supplementary figure 2) to the 1000 Human Genome Ensemble for the same populations () it shows that the published percentage allele count is almost identical to ours (Supplementary Figure 1). Thus, the allele count of the IGF-II rs680 in our sample size is comparable to the allele count in the reference populations where the samples range from 181-379. This is important, because it strengthen the significance of our results even though our sample size is limited.

## Figures and Tables

**Table 1:** 

Sample type	IGF-II ApaI allelic usage	Pathological diagnosis	Surg stage	Grade
Tumor	1M (Het)	Infiltrating ductal carcinoma	II A	3
Normal	2N (Het)	Normal adjacent tissues	II A	3

Tumor	3M (Hom)	Infiltrating ductal carcinoma	II A	3
Normal	4N (Hom)	Normal adjacent tissues	II A	3

Tumor	5M (Het)	Infiltrating ductal carcinoma	II A	3
Normal	6N (Het)	Normal adjacent tissues	II A	3

Tumor	7M (Hom SNP)	Infiltrating ductal carcinoma	III A	
Normal	8N (Hom SNP)	Normal adjacent tissues	III A	

Tumor	9M (Het)	Intraductal carcinoma with microinvasion	IV	3
Normal	10N (Het)	Normal adjacent tissues	IV	3

Tumor	11M (Het)	Infiltrating ductal carcinoma	III A	2
Normal	12N (Het)	Normal adjacent tissues	III A	2

Tumor	13M (Het)	Infiltrating ductal carcinoma	II A	3
Normal	14N (Het)	Normal adjacent tissues	II A	

Tumor	15M (Het)	Infiltrating ductal carcinoma	III A	3
Normal	16N (Het)	Normal adjacent tissues (adenosis)	III A	3

Tumor	17M (Het)	Infiltrating ductal carcinoma	II A	3
Normal	18N (Het)	Normal adjacent tissues	II A	3

Tumor	19M (Hom)	Infiltrating ductal carcinoma	II A	3
Normal	20N (Hom)	Normal adjacent tissues	II A	

Tumor	21M (Het)	Infiltrating ductal carcinoma	III A	3
Normal	22N (Het)	Normal adjacent tissues	III A	3

Tumor	23M (Hom)	Infiltrating ductal carcinoma	IIB	3
Normal	24N (Hom)	Normal adjacent tissues	IIB	3

Tumor	25M (Hom SNP)	Infiltrating ductal carcinoma	III A	3
Normal	26N (Hom SNP)	Normal adjacent tissues	III A	3

Tumor	27M (Hom)	Infiltrating ductal carcinoma	III A	3
Normal	28N (Hom)	Normal adjacent tissues	III A	3

Tumor	29M (Hom SNP)	Infiltrating ductal carcinoma	II	3
Normal	30N (Hom SNP)	Normal adjacent tissues	II	3

Tumor	31M (Hom)	Infiltrating ductal carcinoma	III A	3
Normal	32N (Hom)	Normal adjacent tissues	III A	3

Tumor	33M (Hom SNP)	Infiltrating ductal carcinoma	II	3
Normal	34N (Hom SNP)	Normal adjacent tissues	II	3

Tumor	35M (Het)	Infiltrating ductal carcinoma	III	3
Normal	36N (Het)	Normal adjacent tissues	III	3

Tumor	37M (Het)	Infiltrating ductal carcinoma	III B	2
Normal	38N (Het)	Normal adjacent tissues	III B	2

Tumor	39M (Het)	Infiltrating ductal carcinoma	II	3
Normal	40N (Het)	Normal adjacent tissues	II	3

Tumor	41M (Hom SNP)	Infiltrating ductal carcinoma	I	3
Normal	42N (Hom SNP)	Normal adjacent tissues	I	3

Tumor	43M (Het)	Infiltrating ductal carcinoma	III A	3
Normal	44N (Het)	Normal adjacent tissues	III A	3

Tumor	45M (Het)	Infiltrating ductal carcinoma	I	3
Normal	46N (Het)	Normal adjacent tissues	I	3

Tumor	47M (Het)	Infiltrating ductal carcinoma	III	3
Normal	48N (Het)	Normal adjacent tissues	III	3

## Abbreviations

AA: African American
CA: Caucasian American
VIET: Vietnamese
TNBC: Triple negative breast cancer
BC: Breast cancer
Hom: Homozygous
Het: Heterozygous
Hom SNP: Homozygous with SNP
BA: Biallelic
MA: Monoallelic with no SNP
MAS: Monoallelic with SNP
IGF-I: Insulin-Like Growth Factor-I
IGF-II: Insulin-Like Growth Factor-II
ESR-*α*: Estrogen Receptor-*α*
ERBB2: Human Epidermal Growth Factor Receptor 2 (Her2)
VEGFR: Vascular Endothelial Growth Factor Receptors
PRLR: Prolactin Receptors
IRA: Insulin Receptor A Isoform
IRB: Insulin Receptor B Isoform
SNP: Single Nucleotide Polymorphism
HSK: Housekeeping gene.
