# Corrigendum to “Expression of Intratumoral IGF-II Is Regulated by the Gene Imprinting Status in Triple Negative Breast Cancer from Vietnamese Patients”

**DOI:** 10.1155/2018/8434297

**Published:** 2018-10-23

**Authors:** Vinodh Kumar Radhakrishnan, Lorraine Christine Hernandez, Kendra Anderson, Qianwei Tan, Marino De León, Daisy D. De León

**Affiliations:** Center for Health Disparities and Molecular Medicine, School of Medicine, Loma Linda University, Loma Linda, CA 92350, USA

In the article titled “Expression of Intratumoral IGF-II Is Regulated by the Gene Imprinting Status in Triple Negative Breast Cancer from Vietnamese Patients” [1], there were errors in Figures 2 and 4. There was lane duplication and undeclared splicing in Figure 2, where the bands used for the sample 11M(BA), 12N(BA) were duplicated as lanes 21M(BA), 22N(BA) and the lanes for 15M(BA), 16N(BA) were triplicated as lanes 17M(BA), 18N(BA) and 39M(BA), 40N(BA). There was duplication in Figure 4, where the Ponceau bands in the right-hand panel of Figure 4(a) were rotated 180 degrees in the right-hand panel of Figure 4(c).

The authors asked to correct the article following an institutional investigation, which concluded that these errors were the result of inadvertent human error while cropping and naming the gels and do not affect the conclusions of the article. The labeled scanned data generated from the Gel Doc system are available as Supplementary Materials (available here). In addition, the authors confirmed that the description of the asterisk symbol “∗” should be added to the legend of Figure 2. The corrected Figures 2 and 4 are shown below.

## Figures and Tables

**Figure 1:**
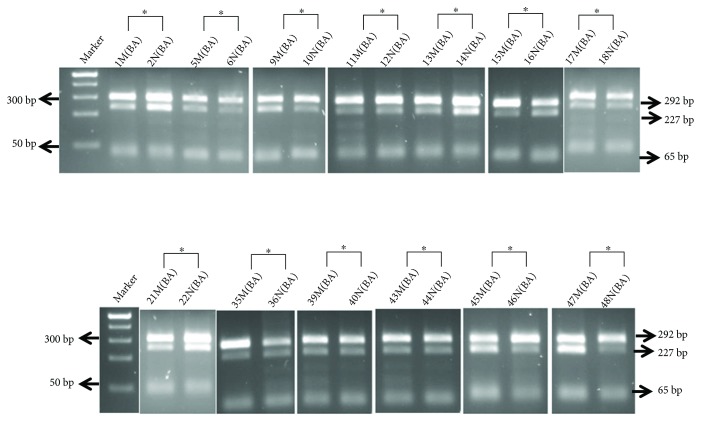
ApaI digestion of the 292 bp nested PCR (QPCR) fragment generated from the fragment of 1.12 RT-PCR reactions. Biallelic expression was identifiable by the presence of each of the 292, 227, and 63 bp restriction fragments. The first lane shows Fisher's exACTGene 100 bp DNA Ladder, showing the 500 bp–25 bp respective molecular weight size markers. ∗ denotes paired breast samples from the same patient (normal, malignant).

**Figure 2:**
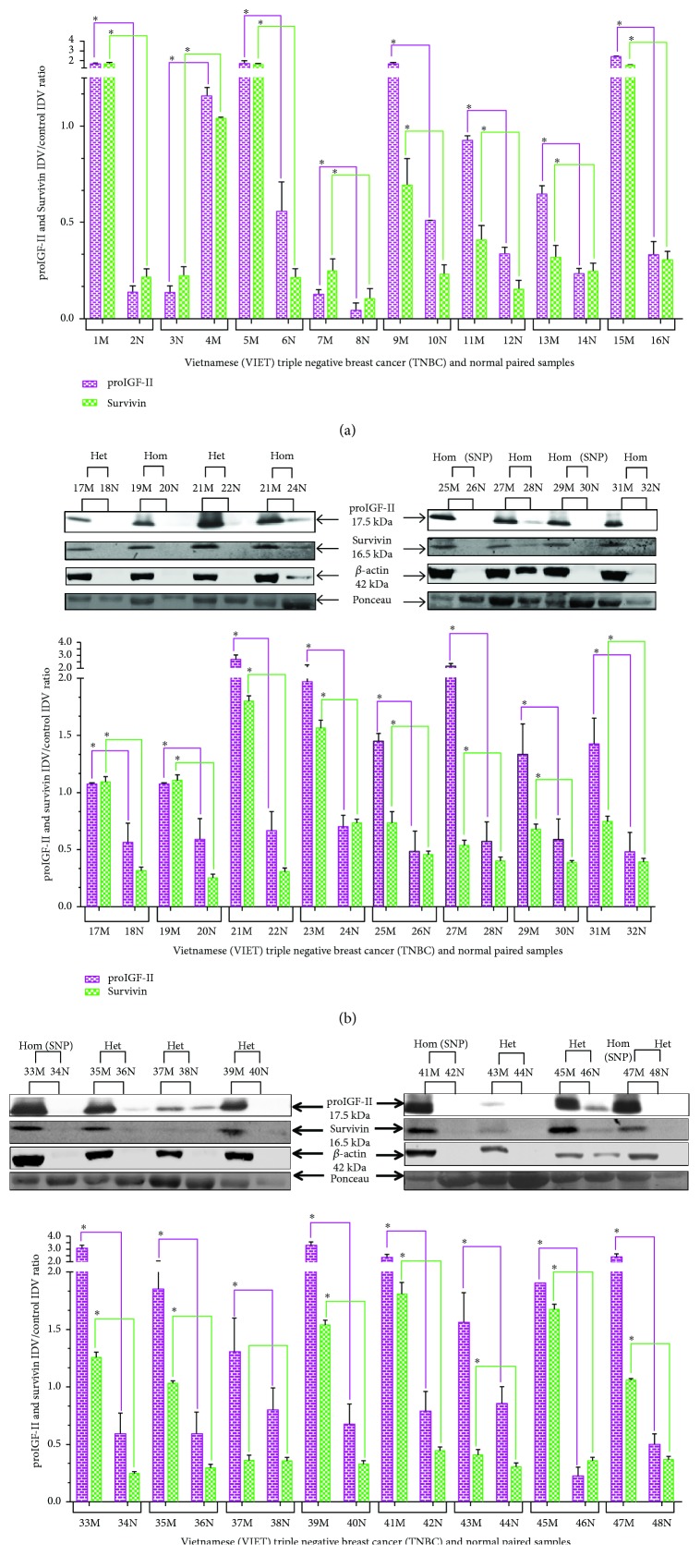
((a), (b), and (c)) Western blot of paired normal/malignant TNBC samples (1–48). The total number of samples (*n*) analyzed per group was as follows: Heterozygous (Het; *n* = 27), Homozygous with SNP (Hom SNP; *n* = 11), and Homozygous (Hom; *n* = 10). Bar graphs (a–c) of free proIGF-II (17.5 kDa) and Survivin (16.5 kDa). Ponceau red staining was used to normalize for sample loading. Bars represent the mean ± SE of all normalized samples per group. Asterisks indicate values statistically significant ∗(*p* < 0.05) using the Wilcoxon paired 𝑡-test.

## Supplementary Materials

Supplementary Material Supplementary Materials: the labeled scanned data generated from the Gel Doc system.
