# Supplementary material for: Expression of Intratumoral IGF-II Is Regulated by the Gene Imprinting Status in Triple Negative Breast Cancer from Vietnamese Patients
Source: Int J Endocrinol. 2015 Sep 10;2015:401851. doi: 10.1155/2015/401851 (PMC4581569; doi:10.1155/2015/401851)
Supplement: Supplementary file 1 — Since our breast cancer samples from Vietnamese patients is limited (n=48), we provided Supplementary Figure 1 which represents the results of the IGF-II rs680 C/T allele count from the 1000 Human Genome Ensemble () from different ethnic groups. Supplementary Figure 2 represents unpublished data from our own IGF-II rs680 C/T allele count from paired breast cancer samples from Caucasian American (n=32), South Korean (n=42) and African American (n=36). Please note that when we compared the allele count for the rs680 SNP in our paired breast sample analysis from Caucasian American, South Korean and African American (Supplementary figure 2) to the 1000 Human Genome Ensemble for the same populations () it shows that the published percentage allele count is almost identical to ours (Supplementary Figure 1). Thus, the allele count of the IGF-II rs680 in our sample size is comparable to the allele count in the reference populations where the samples range from 181-379. This is important, because it strengthen the significance of our results even though our sample size is limited. [file 401851.f1.docx]

**Supplementary: 1000 genome phase 1 ensemble human genome population studies**

**
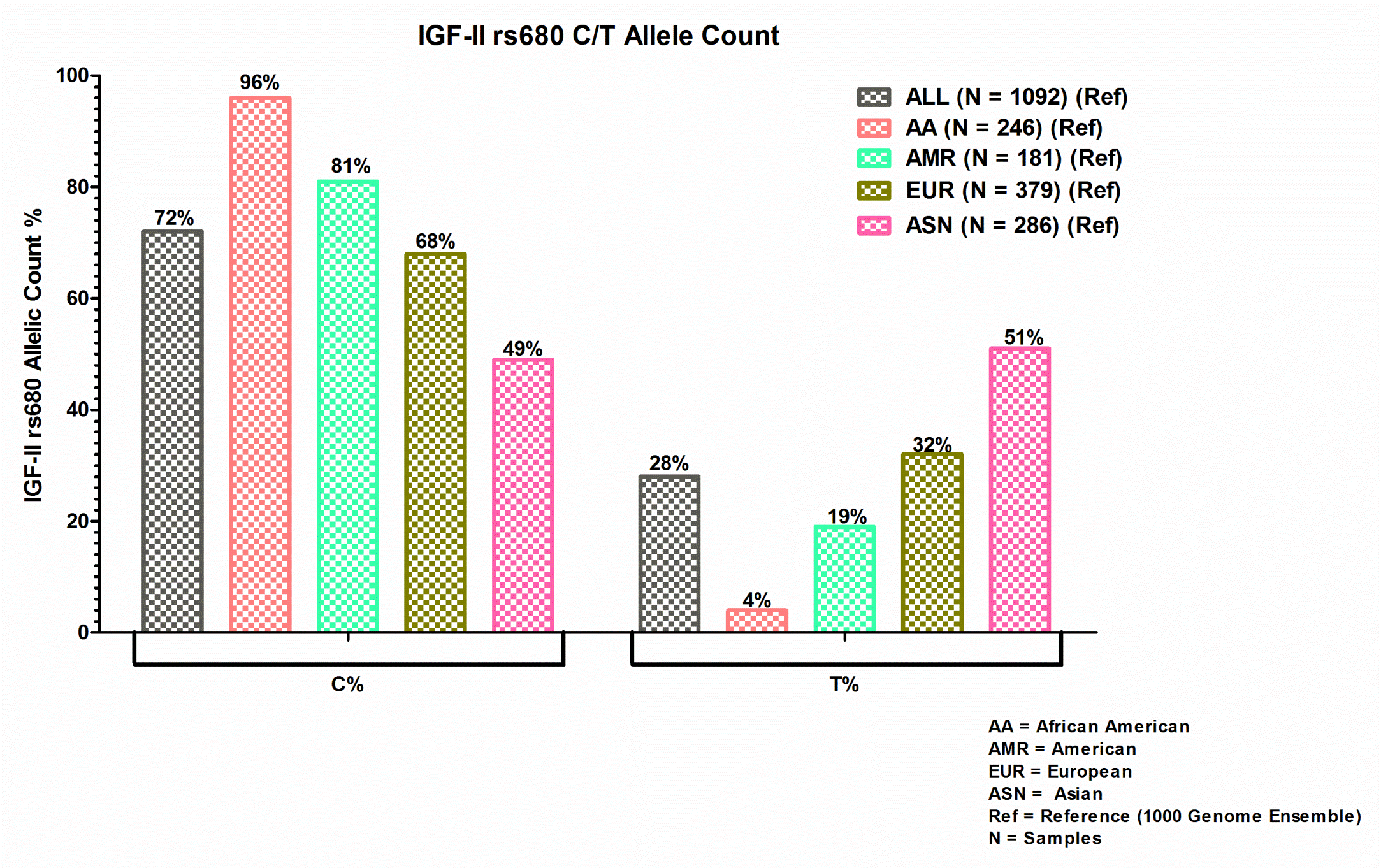
**

**S. Figure 1**

Analysis include total reference samples All (N = 1092), African American (AA) (N=246), American (AMR) (N=181), European (EUR) (N= 379), Asian (ASN) (N= 286). C/T IGF-II rs680 allelic count information is obtained from the 1000 genome phase 1 ensemble human genome population studies. Bar graph shows the representation information of the allelic count % for IGF-II rs680 observed among men and women population across different ethnic groups.

**Supplementary: Limited Breast Sample Analysis of IGF-II rs680 in Different Ethnic Groups**

**
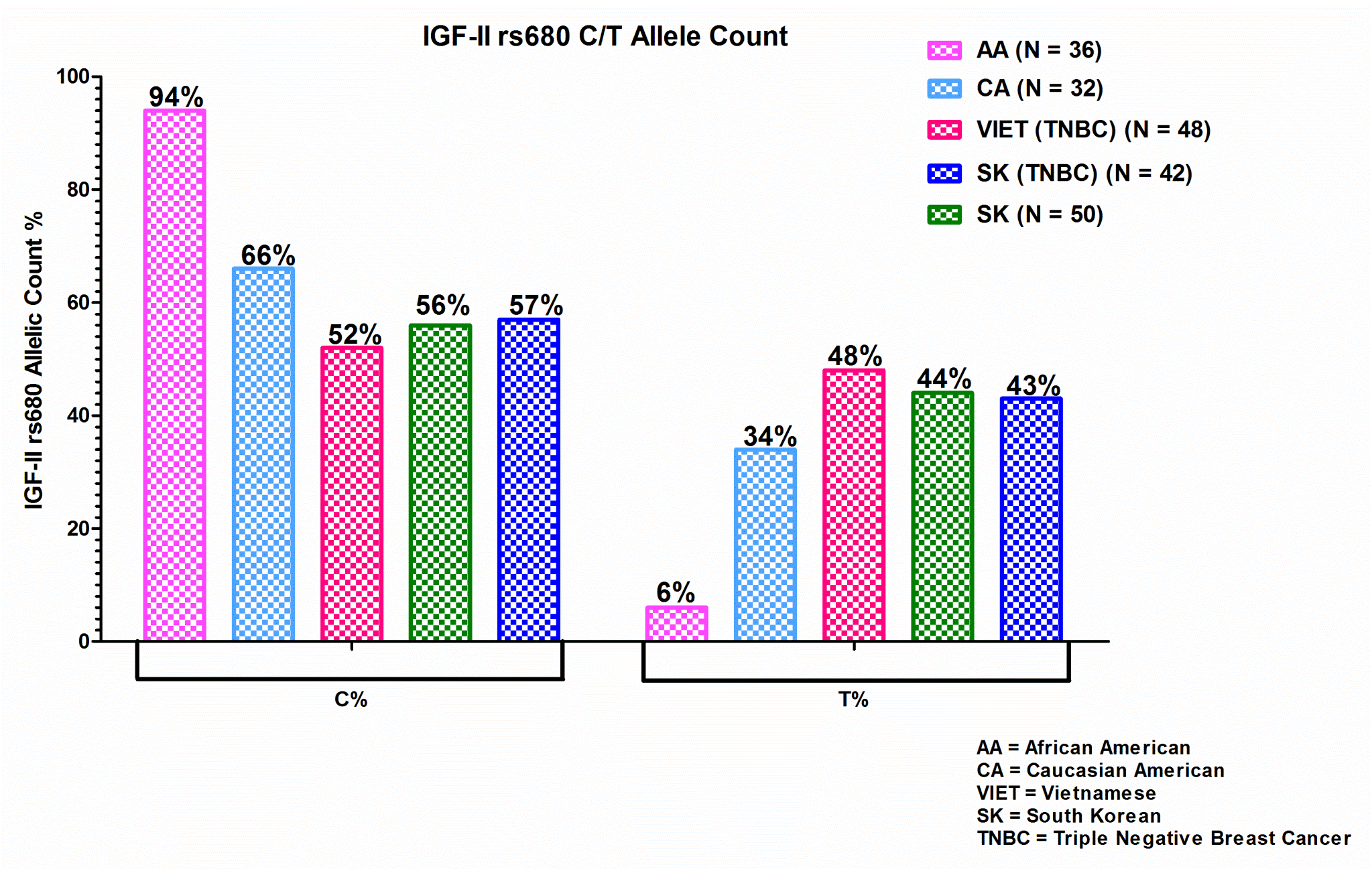
**

**S. Figure 2**

Analysis include experimental samples African American (AA) (N=36), Caucasian American (CA) (N=32), Vietnamese (VIET) (N=48) TNBC, South Korean (SK) (N= 50), South Korean (SK) (N= 42) TNBC. C/T IGF-II rs680 allelic count information are calculated as per the 1000 genome phase 1 ensemble human genome rs680 SNP variation studies. Bar graph shows the representation of the allelic count information % for IGF-II rs680 SNP observed in breast cancer and adjacent normal samples from women of different ethnic groups analyzed in our breast cancer laboratory.
