# Supplementary material for: Corrigendum to “Expression of Intratumoral IGF-II Is Regulated by the Gene Imprinting Status in Triple Negative Breast Cancer from Vietnamese Patients”
Source: Int J Endocrinol. 2018 Oct 23;2018:8434297. doi: 10.1155/2018/8434297 (PMC6218738; doi:10.1155/2018/8434297)
Supplement: Supplementary Material — s: the labeled scanned data generated from the Gel Doc system. [file 8434297.f1.pptx]

## Slide 1
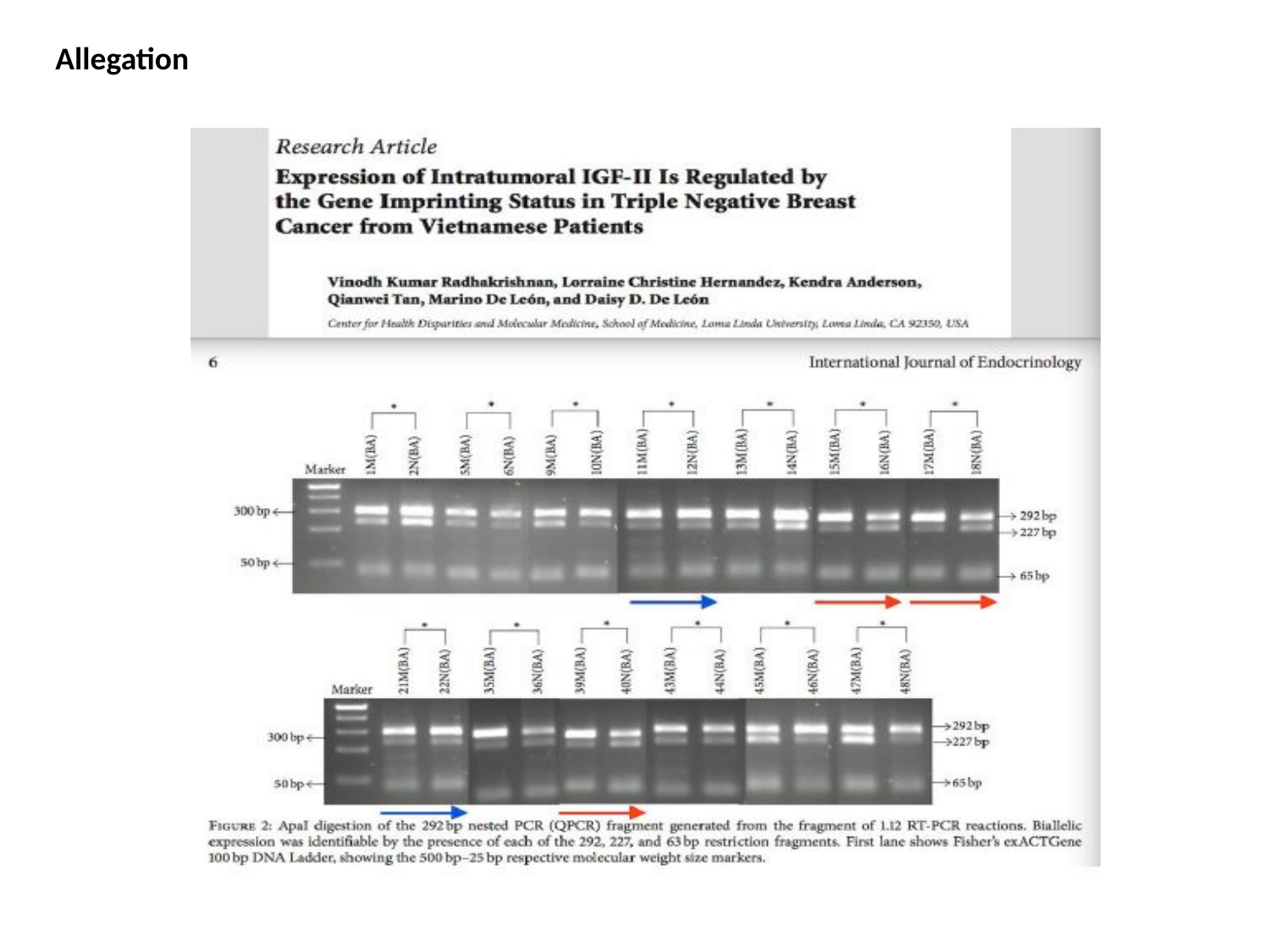

Allegation

## Slide 2
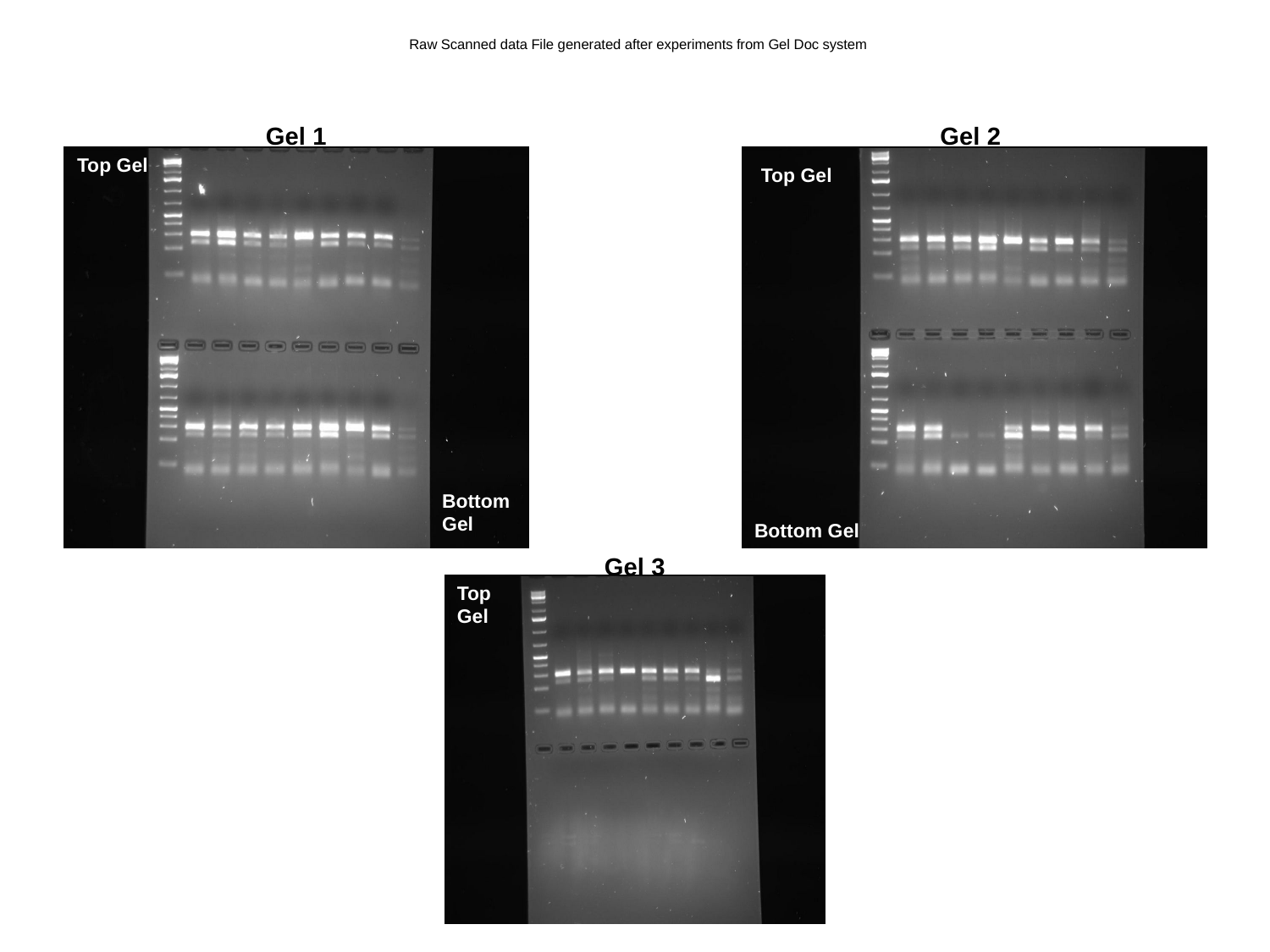

# Raw Scanned data File generated after experiments from Gel Doc system
Gel 2
Gel 1
Top Gel
Top Gel
Bottom
Gel
Bottom Gel
Gel 3
Top Gel

## Slide 3
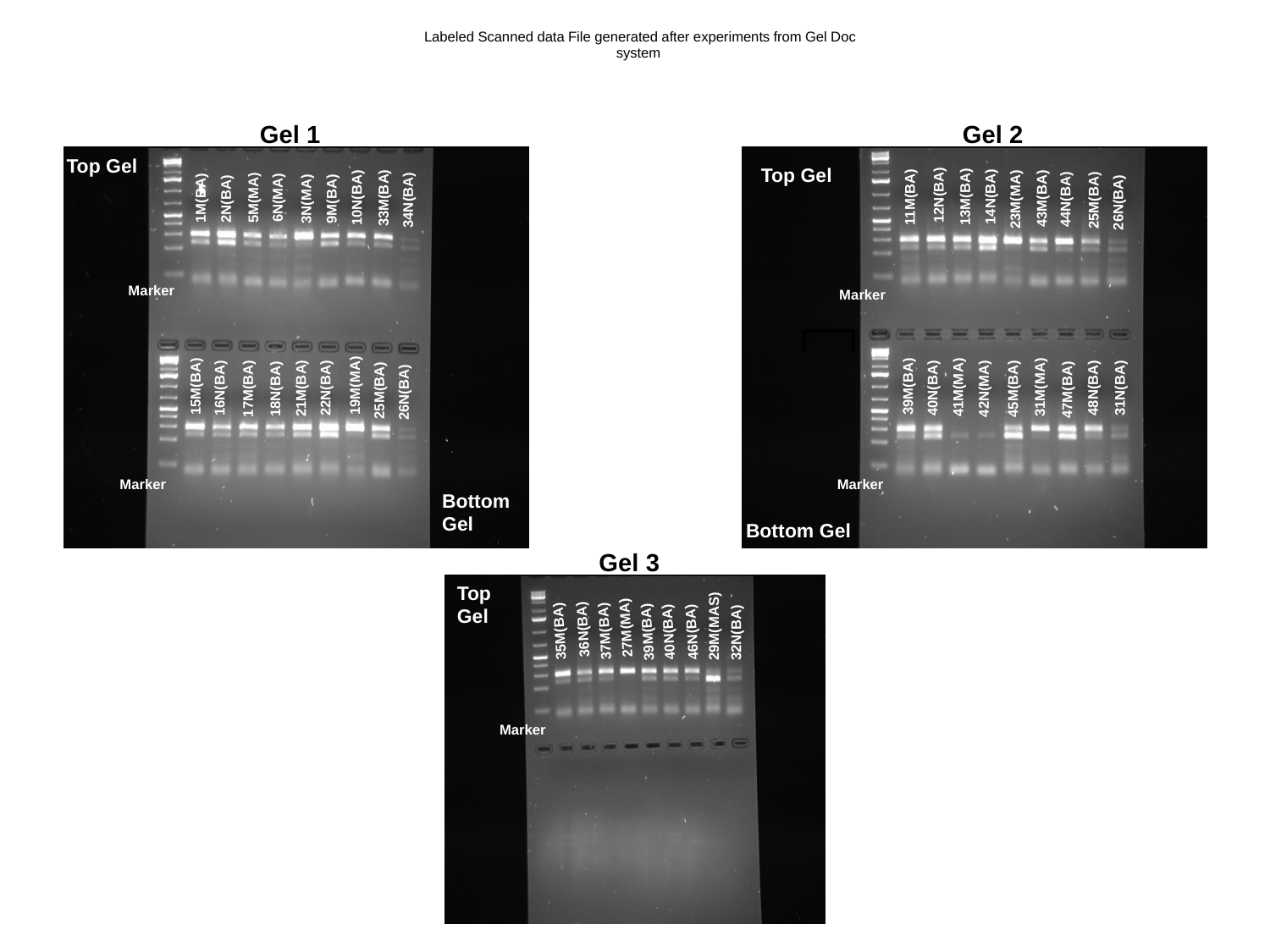

# Labeled Scanned data File generated after experiments from Gel Doc system
Gel 1
Gel 2
Gel 2
Top Gel
Marker
Top Gel
12N(BA)
14N(BA)
13M(BA)
11M(BA)
6N(MA)
5M(MA)
33M(BA)
10N(BA)
1M(BA)
43M(BA)
2N(BA)
9M(BA)
3N(MA)
23M(MA)
44N(BA)
34N(BA)
25M(BA)
26N(BA)
Marker
Marker
19M(MA)
39M(BA)
15M(BA)
31M(MA)
41M(MA)
48N(BA)
40N(BA)
16N(BA)
21M(BA)
22N(BA)
31N(BA)
17M(BA)
42N(MA)
45M(BA)
18N(BA)
47M(BA)
25M(BA)
26N(BA)
Marker
Marker
Bottom
Gel
Bottom Gel
Gel 3
Top Gel
29M(MAS)
27M(MA)
36N(BA)
35M(BA)
37M(BA)
39M(BA)
40N(BA)
46N(BA)
32N(BA)
Marker

## Slide 4
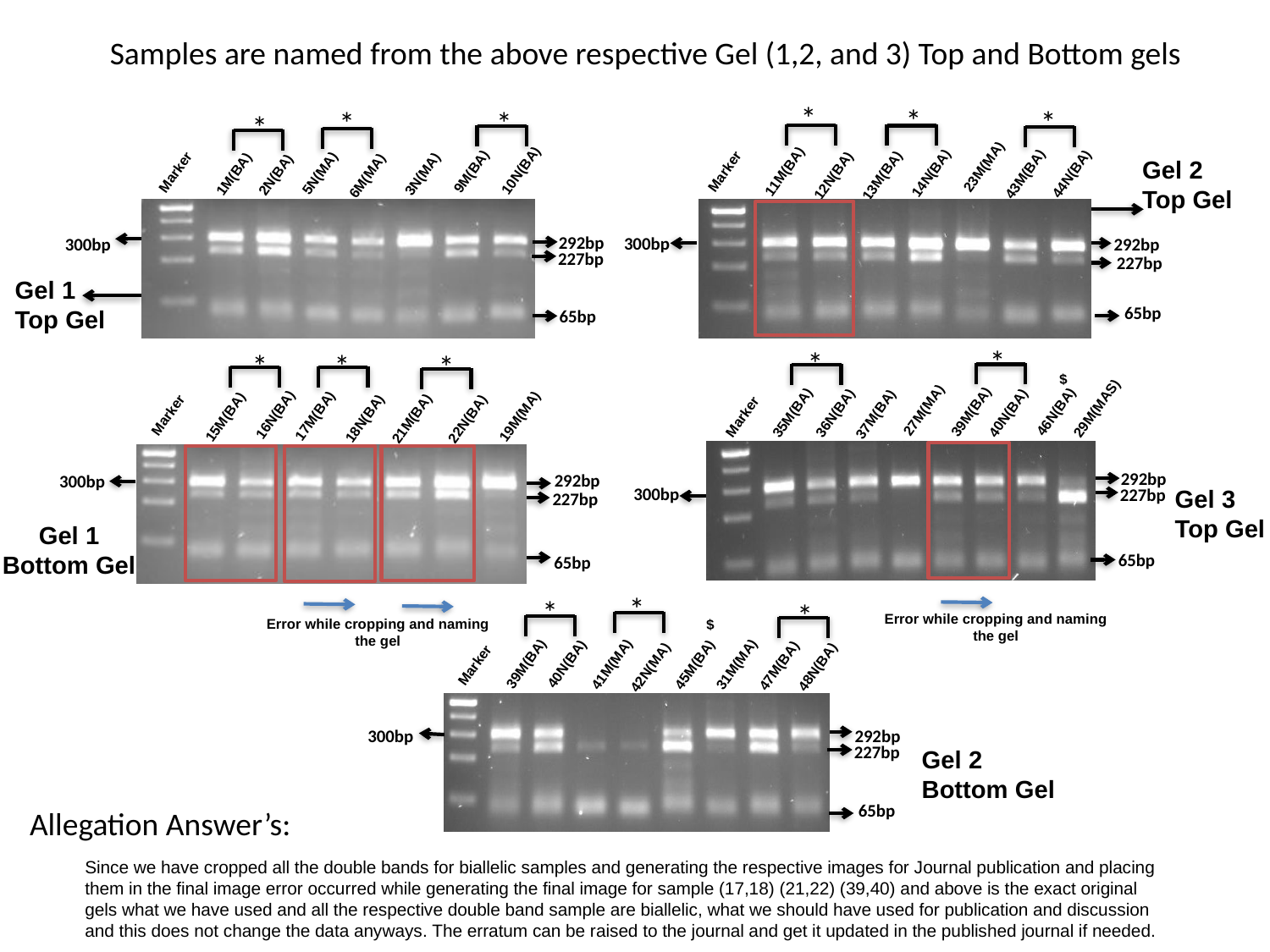

Samples are named from the above respective Gel (1,2, and 3) Top and Bottom gels
*
*
*
*
*
*
Gel 2
Top Gel
23M(MA)
10N(BA)
9M(BA)
11M(BA)
Marker
Marker
5N(MA)
14N(BA)
44N(BA)
3N(MA)
1M(BA)
43M(BA)
2N(BA)
12N(BA)
13M(BA)
6M(MA)
292bp
300bp
300bp
292bp
227bp
227bp
Gel 1
Top Gel
65bp
65bp
*
*
*
*
*
$
29M(MAS)
27M(MA)
46N(BA)
39M(BA)
35M(BA)
36N(BA)
40N(BA)
37M(BA)
16N(BA)
Marker
17M(BA)
19M(MA)
Marker
15M(BA)
18N(BA)
21M(BA)
22N(BA)
292bp
292bp
300bp
300bp
Gel 3
Top Gel
227bp
227bp
Gel 1
Bottom Gel
65bp
65bp
*
*
*
Error while cropping and naming
the gel
Error while cropping and naming
the gel
$
39M(BA)
40N(BA)
41M(MA)
45M(BA)
31M(MA)
Marker
47M(BA)
48N(BA)
42N(MA)
300bp
292bp
227bp
Gel 2
Bottom Gel
292bp
65bp
Allegation Answer’s:
227bp
Since we have cropped all the double bands for biallelic samples and generating the respective images for Journal publication and placing them in the final image error occurred while generating the final image for sample (17,18) (21,22) (39,40) and above is the exact original gels what we have used and all the respective double band sample are biallelic, what we should have used for publication and discussion and this does not change the data anyways. The erratum can be raised to the journal and get it updated in the published journal if needed.
65bp

## Slide 5
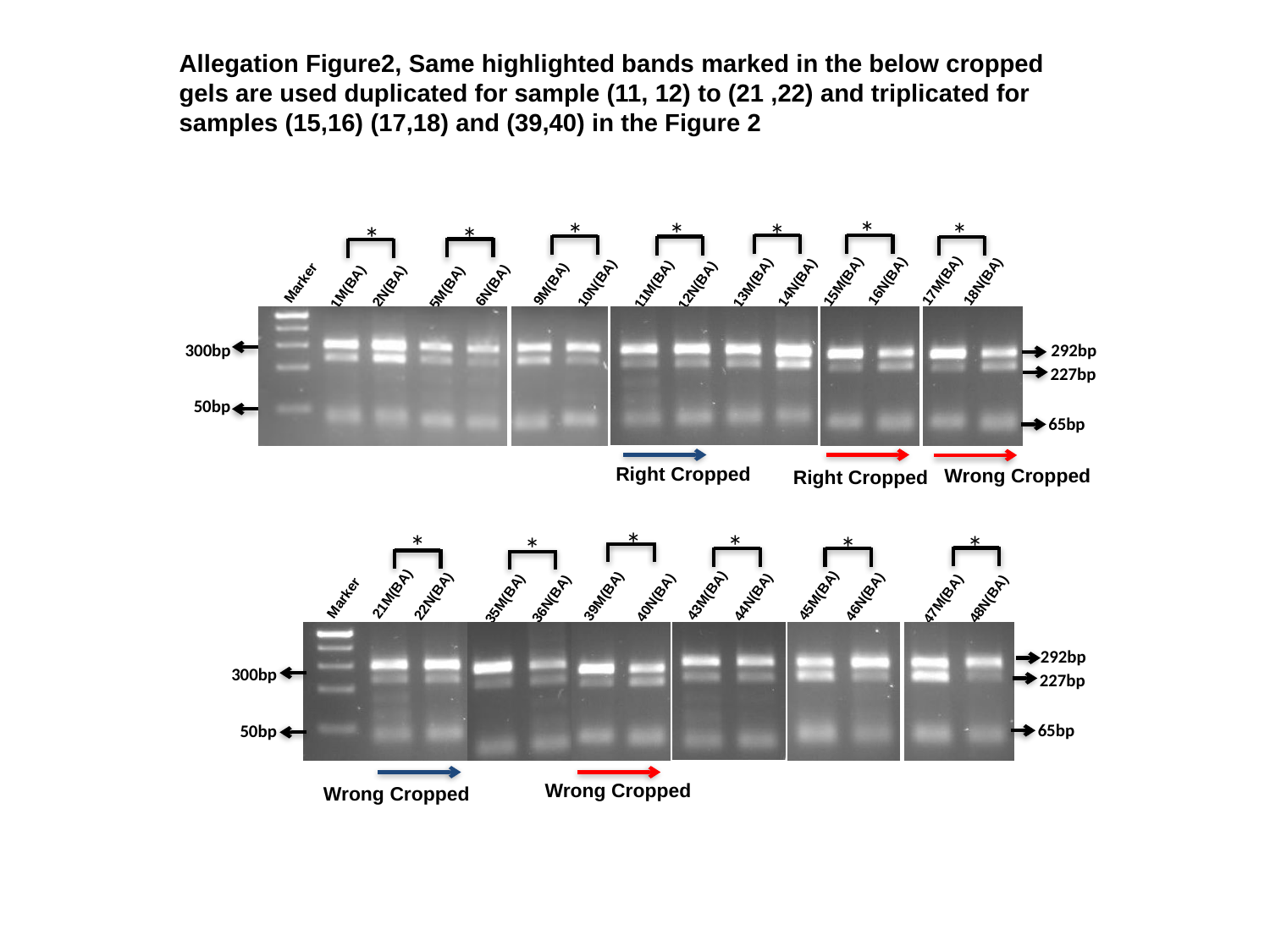

Allegation Figure2, Same highlighted bands marked in the below cropped gels are used duplicated for sample (11, 12) to (21 ,22) and triplicated for
samples (15,16) (17,18) and (39,40) in the Figure 2
*
*
*
*
*
*
*
17M(BA)
16N(BA)
18N(BA)
15M(BA)
Marker
14N(BA)
13M(BA)
10N(BA)
9M(BA)
11M(BA)
12N(BA)
6N(BA)
2N(BA)
1M(BA)
5M(BA)
300bp
292bp
227bp
50bp
65bp
Right Cropped
Wrong Cropped
Right Cropped
*
*
*
*
*
*
21M(BA)
45M(BA)
43M(BA)
22N(BA)
39M(BA)
46N(BA)
44N(BA)
40N(BA)
Marker
35M(BA)
36N(BA)
48N(BA)
47M(BA)
292bp
300bp
227bp
65bp
50bp
Wrong Cropped
Wrong Cropped
